# Progesterone for Acute Traumatic Brain Injury: A Systematic Review of Randomized Controlled Trials

**DOI:** 10.1371/journal.pone.0140624

**Published:** 2015-10-16

**Authors:** Yunhui Zeng, Yujie Zhang, Junpeng Ma, Jianguo Xu

**Affiliations:** 1 Department of Neurosurgery, West China Hospital, West China Medical School, Sichuan University, Chengdu, Sichuan, People’s Republic of China; 2 The Second Integrated Unit, West China Hospital, West China Medical School, Sichuan University, Chengdu, Sichuan, People’s Republic of China; Scientific Inst. S. Raffaele Hosp., ITALY

## Abstract

**Objective:**

To evaluate the efficacy and safety of progesterone administrated in patients with acute traumatic brain injury (TBI).

**Methods:**

PubMed/MEDLINE, EMBASE, Cochrane Database of Systematic Reviews, Cochrane Central Register of Controlled Trials (CENTRAL), Clinicaltrials.gov, ISRCTN registry and WHO International Clinical Trials Registry Platform (ICTRP) were searched for randomized controlled trials (RCTs) comparing progesterone and placebo administrated in acute TBI patients. The primary outcome was mortality and the secondary outcomes were unfavorable outcomes and adverse events. A meta-analysis was conducted to evaluate the efficacy and safety of progesterone administrated in patients with acute TBI.

**Results:**

A total of 6 studies met inclusion criteria, involving 2,476 patients. The risk of bias was considered to be low in 4 studies but high in the other 2 studies. The results of meta-analysis indicated progesterone did not reduce the mortality (RR = 0.83, 95% CI = 0.57–1.20) or unfavorable outcomes (RR = 0.89, 95% CI = 0.78–1.02) of acute TBI patients in comparison with placebo. Sensitivity analysis yielded consistent results. Progesterone was basically safe and well tolerated in TBI patients with the exception of increased risk of phlebitis or thrombophlebitis (RR = 3.03, 95% CI = 1.96–4.66).

**Conclusions:**

Despite some modest bias, present evidence demonstrated that progesterone was well tolerated but did not reduce the mortality or unfavorable outcomes of adult patients with acute TBI.

## Introduction

Traumatic brain injury (TBI) is one of the leading causes of mortality and disability worldwide especially among young adults, which exerts great influence on human health and social economy [1]. More than 1.7 million people experience a TBI every year in the United States and around 5.3 million people are living with a lifelong disability related to TBI [2, 3]. With increased use of motor vehicles, the incidence of TBI is increasing worldwide, particularly in developing countries [4]. However, the TBI-related mortality has not decreased significantly and the recovery outcome of TBI has not improved much over the past two decades, partially due to the lack of effective treatment strategies [5, 6]. It is urgent to find out a safe and effective therapy to improve the outcome of TBI patients.

Progesterone, a potent neurosteroid synthesized in the central nervous system, is one of the promising drug candidates for treatment of acute TBI [6]. A plenty of experimental studies investigated the impact of progesterone on central nervous system with various animal models, and growing evidence suggested that progesterone exerted neuroprotective properties by decreasing vasogenic cerebral edema, protecting and rebuilding the blood-brain barrier, improving neuronal survival, modulating the inflammatory cascade and limiting cellular necrosis and apoptosis after acute TBI [7–9]. Based on the encouraging preliminary outcomes, a series of clinical trials were conducted to evaluate the efficacy and safety of progesterone administrated in patients with TBI [10–16]. The limited evidence from a Cochrane systematic review published in 2012 revealed that progesterone might improve the neurologic outcome of acute TBI patients [17]. However, the results of two phase III multicenter randomized controlled trials (RCTs) were released and suggested that acute TBI patients unexpectedly did not benefit from progesterone administration, which attracted broad attention and extensive discussion [18–23]. With the opposite conclusions drawn in previous clinical trials, the exact effect of progesterone on TBI patients was confused. Therefore, it is necessary to reanalyze the results of previous and newly published RCTs and perform a systematic review to evaluate the efficacy and safety of progesterone in comparison with placebo administrated in patients with acute TBI.

## Methods

### Searching Strategy

The literature retrieval was aimed to identify all eligible studies that evaluated the efficacy and safety of progesterone in comparison with placebo administrated in patients with TBI. Electronic databases of PubMed/MEDLINE, EMBASE, Cochrane Database of Systematic Reviews, Cochrane Central Register of Controlled Trials (CENTRAL), Clinicaltrials.gov, ISRCTN registry and WHO International Clinical Trials Registry Platform (ICTRP) were searched without language restriction from database inception to March 27, 2015. The keywords “progesterone”, “progestin”, “traumatic brain injury”, “TBI”, “head injury” and “brain trauma” were used in various combinations. The reference lists of all included studies and reviews were also searched manually as a complement to the computer searches.

### Study Selection

Two independent reviewers screened the titles and abstracts of primary identified studies for eligibility. Full-text articles were read for further assessment if the eligibility was unclear by screening the abstracts. Any discrepancy in the eligibility was resolved through discussion by the review team.

Several inclusion criteria were used to select eligible studies: (1) Published and unpublished RCTs comparing progesterone versus placebo administrated in patients with acute TBI; (2) Patients with clinically diagnosis of acute TBI of any severities secondary to head injury; (3) Progesterone or placebo treatment started within 24 hours of the head injury regardless of administration route, dose or duration. Excluded criteria included: (1) Full-text published in other languages rather than English or Chinese because of resource limitation; (2) Studies using synthetic progestin rather than progesterone as intervention; (3) Studies pertaining to overlapping patients of other included studies. Only progesterone was considered as the intervention in the systematic review and meta-analysis because the biochemical characteristics of synthetic progestin were not equivalent to natural progesterone in post-injury treatment [24, 25].For preliminary included studies, names of all authors and the medical centers involved were examined carefully to avoid duplication data. Whenever studies pertained to overlapping patients, studies with larger sample size and more comprehensive data were retained.

### Data Extraction

Two independent reviewers extracted the details of included studies with a standardized form, including sequence generation, allocation concealment, blinding methods, demographic characteristics of participants, types of interventions, original data of results, follow-up period, methods of analysis (intention-to-treat analysis or per protocol analysis, or both), comparability of groups at baseline and statistical methods. The primary and secondary outcomes of interest for the meta-analysis were mortality and unfavorable outcome at the end of follow-up period. As described in the former Cochrane systematic review, we divided the Glasgow Outcome Scale (GOS) and Extended Glasgow Outcome Scale (GOS-E) scores into favorable (moderate disability or good recovery, i.e. GOS 4 to 5, or GOS-E 5 to 8) and unfavorable outcomes (death, vegetative state or severe disability, i.e. GOS 1 to 3, or GOS-E 1 to 4) [17]. Adverse events were also collected as secondary outcomes to perform descriptive evaluation. We tried to contact with authors for more information if necessary. The data extracted by each reviewer were compared and any disagreement was resolved by discussion.

### Quality Assessment

The risk of bias in included studies was assessed by two independent reviewers with the Cochrane risk-of-bias tool described in the Cochrane Handbook for Systematic Reviews of Interventions version 5.1.0 [26]. The risk of selection bias (random sequence generation and allocation concealment), performance bias (blinding of participants and personnel), detection bias (blinding of outcome assessment), attrition bias (incomplete outcome data), reporting bias (selective reporting) and other bias were judged respectively to be “Low risk”, “High risk” or “Unclear risk”. We reviewed the full-text, supplementary information and protocols to assess the qualities of included RCTs. Any discrepancy about the judgment was resolved by discussion of the review team.

### Data Synthesis and Analysis

The relative ratio (RR) and 95% confidence interval (95% CI) of mortality and unfavorable outcomes at the end of follow-up period were used to estimate the efficacy of progesterone. The pooled RR<1 indicated that progesterone improved the outcomes of patients with acute TBI and the results were considered significant when the 95% CI did not overlap 1.

The p value of Heterogeneity chi-squared (χ^2^) test and I-squared (I^2^) value were calculated to assess the heterogeneity of the included studies. Given the limited number of eligible studies, the RRs of each study were synthesized in a random effect meta-analysis using DerSimonian-Laird algorithm. The potential publication bias on results was assessed by Begg’s test and Egger’s test, with the significant level of p<0.05 [27, 28]. Heterogeneity tests, meta-analyses and tests for publication bias were all carried out with software STATA version 11.0 (Stata Corporation, College Station, TX, USA).

### Sensitivity and Subgroup Analyses

According to the quality assessment, sensitivity analysis was performed using studies with low risk of bias to evaluate the reliability of the results of meta-analysis and determine the potential impact of studies of poor qualities on the results of meta-analysis. Subgroup analysis was performed according to the severity of TBI and therapeutic regimen. The severity of TBI was measured by Glasgow Coma Scale (GCS) scores, including severe (GCS< = 8) and moderate (GCS 9 to 12) TBI subgroups. The therapeutic regimen of progesterone included intravenous and intramuscular route at different dose.

## Results

### Searching Results and Characteristics of Included Studies

**Fig 1:**
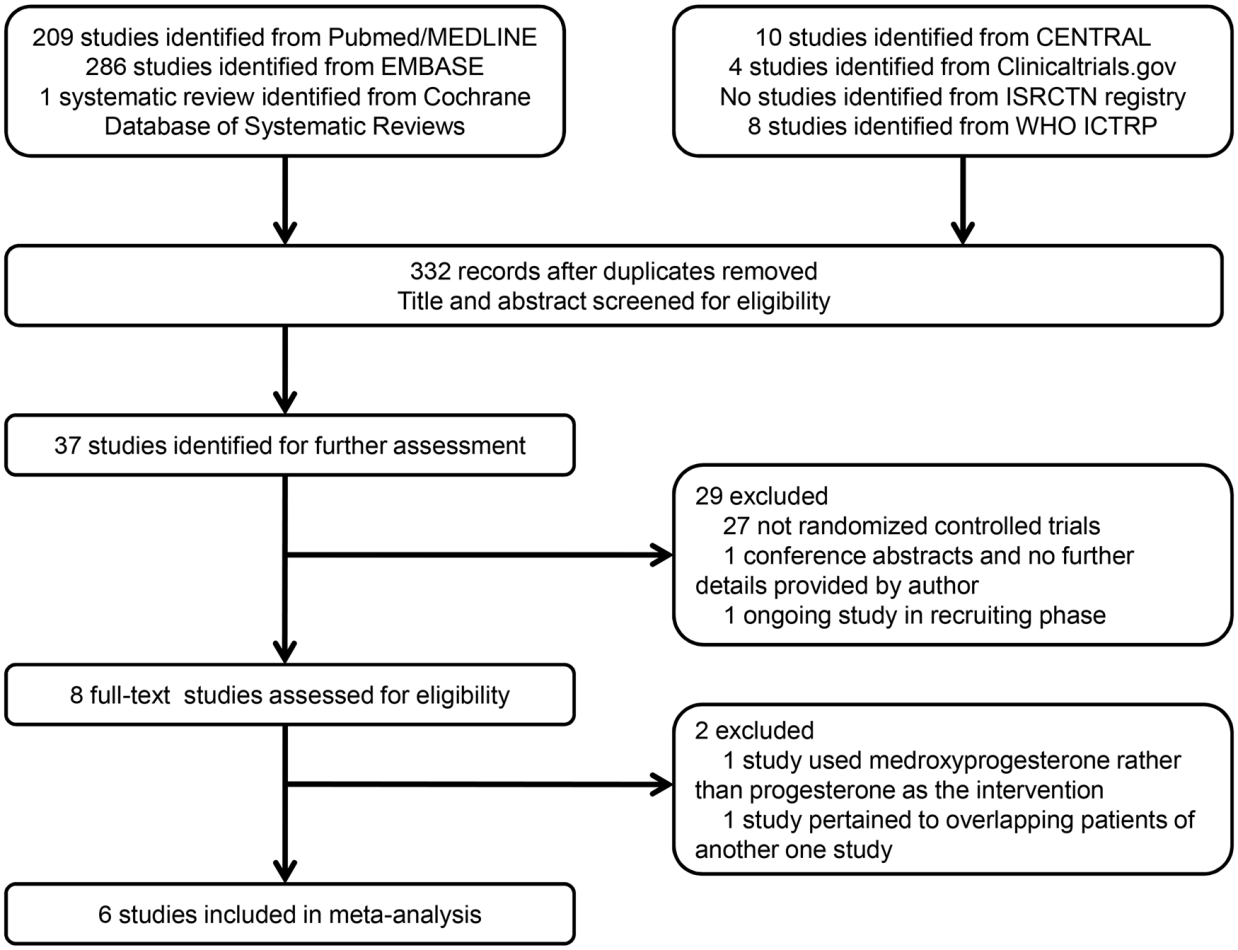
Flow diagram showing selection of studies.

A total of 332 studies were initially identified after duplicates removed through the prespecified search strategy, and 37 studies were retrieved for full-text after abstract screening. There were 1 ongoing study in recruiting phase and 1 conference abstract without sufficient information and therefore they were excluded [16, 29]. Another 27 studies were excluded because they were not randomized controlled trials. After full-text reading, 1 study included in the former Cochrane systematic review was excluded, because the participants recruited from March 2003 to December 2005 were considered to overlap those of another study that were recruited from March 2003 to February 2007 at the same hospital [10]. According to the inclusion criteria, one more study was excluded because the intervention was medroxyprogesterone rather than natural progesterone [15]. Finally, a total of 6 studies met all the inclusion criteria and were included in meta-analysis as shown in Fig 1 [11–14, 18, 19].

The included studies were described in Table 1. Six RCTs with 2476 patients were identified from 2007 to 2014. The recruiting time was from May 2002 to October 2013. The sample sizes ranged from 40 to 1195 and male accounted for 65.3%. Participants were all adult with the age of 16 to 94 years old. The participants of 4 studies were patients with severe TBI (GCS < = 8) while those of the other 2 studies were patients with moderate or severe TBI (GCS 4 to 12). Progesterone was administrated intravenously at a dose of 0.5–0.71 mg/kg or intramuscularly at 1.0 mg/kg. Progesterone was administrated for 3 to 5 days. Placebo was used in all the 6 RCTs. The follow-up period varied from 1 to 6 months. Outcome measures included mortality, GOS/GOS-E, intracranial pressure (ICP), body temperature, blood pressure, adverse events and so on.

**Table 1:** Main characteristics of the 6 included RCTs.

Study	Year	Country	Sample size (male/%)	Age/y	Participants	Intervention	Follow-up	Outcome measures
**Wright (11)**	2007	USA	100(71)	>18	GCS score of 4 to 12	Intravenously 0.71 mg/kg progesterone for the first hour and 0.5 mg/kg per hour for the next 71 hours.	1 month	Mortality, Dichotomized GOS, DRS; Duration of coma, Duration of post-traumatic amnesia at 30 days post-injury; ICP, temperature, blood pressure during the first 3 days of treatment and for 1 day afterwards. Adverse events.
**Xiao (12)**	2008	China	159(72)	18 to 65	GCS score of ≤ 8	Intramuscularly 1.0 mg/kg progesterone every 12 hours for 5 consecutive days.	6 months	Mortality, GOS and Modified Functional Independence Measure scores at 3 and 6 months after injury. ICP, average body temperature during treatment. Complications and adverse events.
**Abokhabar (13)**	2012	Egypt	100(NR)	NR	GCS score of ≤ 8	Intramuscularly 1.0 mg/kg progesterone every 12 hours for 5 consecutive days.	1 month	GOS at 30-day after injury; Duration of ICU stay
**Aminmansour (14)**	2012	Iran	40(70)	29.78*	GCS < 8	Intramuscularly 1.0 mg/kg progesterone every 12 hours for 5 consecutive days.	3 months	GCS during hospitalization and 1 month after treatment; GOS after 3 months.
**Skolnick (18)**	2014	Asia, Europe, North and South America	1195(79)	16 to 70	GCS score ≤ 8	Intravenously 0.71 mg/kg progesterone for the first hour and 0.5 mg/kg per hour for the next 119 hours.	6 months	GOS and GOS-E score at 3 and 6 months after the injury; Mortality at 1 month and 6 months; Changes in ICP, cerebral perfusion pressure, therapeutic intensity levels, intracranial pathologic findings on day 6, and SF-36 scale at 3 and 6 months.
**Wright (19)**	2014	USA	882(74)	17 to 94	GCS score of 4 to 12	Intravenously 0.71 mg/kg progesterone for the first hour, 0.50mg/kg for the next 71 hours and tapered by 0.125 mg/kg every 8 hours, for a total of 96 hours.	6 months	GOS-E at 6 months; Mortality, the Disability Rating Scale score, adverse events; cognitive, psychological and neurologic outcomes.

NOTE: y = year; TBI = traumatic brain injury; GCS = Glasgow Coma Scale; GOS = Glasgow Outcome Scale; DRS = Disability Rating Score; ICP = intracranial pressure; NR = not reported; ICU = intensive care unit; GOS-E = Extended Glasgow Outcome Scale; SF-36 = 36-Item Short-Form Health Survey;

*mean age.

### Quality Assessment of Included Studies

According to “Table 8.5.d: Criteria for judging risk of bias” in Cochrane Handbook for Systematic Reviews of Interventions version 5.1.0, the risk of bias in included studies was assessed by two independent reviewers [26]. As shown in Table 2, the risk of bias was considered to be low in 4 studies and high in the other 2 studies. For one study, only the preliminary results was published as a conference abstract and no further information was available to assess the quality despite of every effort in contacting the authors [13]. For the other study, randomization techniques, allocation concealment or blinding method were underreporting in the article, and no protocol was available to judge the risk of other bias [14]. Based on the assessment, sensitivity analysis was performed with the 4 studies with low risk of bias.

**Table 2:** Quality assessment of the 6 RCTswith the Cochrane risk-of-bias tool.

Study	Random sequence generation	Allocation concealment	Blinding of participants and personnel	Blinding of outcome assessment	Incomplete outcome data	Selective reporting	Other bias
**Wright 2007 (11)**	Low risk	Low risk	Low risk	Low risk	Low risk	Low risk	Low risk
**Xiao 2008 (12)**	Low risk	Low risk	Low risk	Low risk	Low risk	Low risk	Low risk
**Abokhabar 2012 (13)**	High risk	High risk	High risk	High risk	Unclear risk	Unclear risk	Unclear risk
**Aminmansour 2012 (14)**	High risk	High risk	High risk	High risk	Low risk	Low risk	Unclear risk
**Skolnick 2014 (18)**	Low risk	Low risk	Low risk	Low risk	Low risk	Low risk	Low risk
**Wright 2014 (19)**	Low risk	Low risk	Low risk	Low risk	Low risk	Low risk	Low risk

### Primary Outcome: Mortality at the End of Follow-up Period

**Fig 2:**
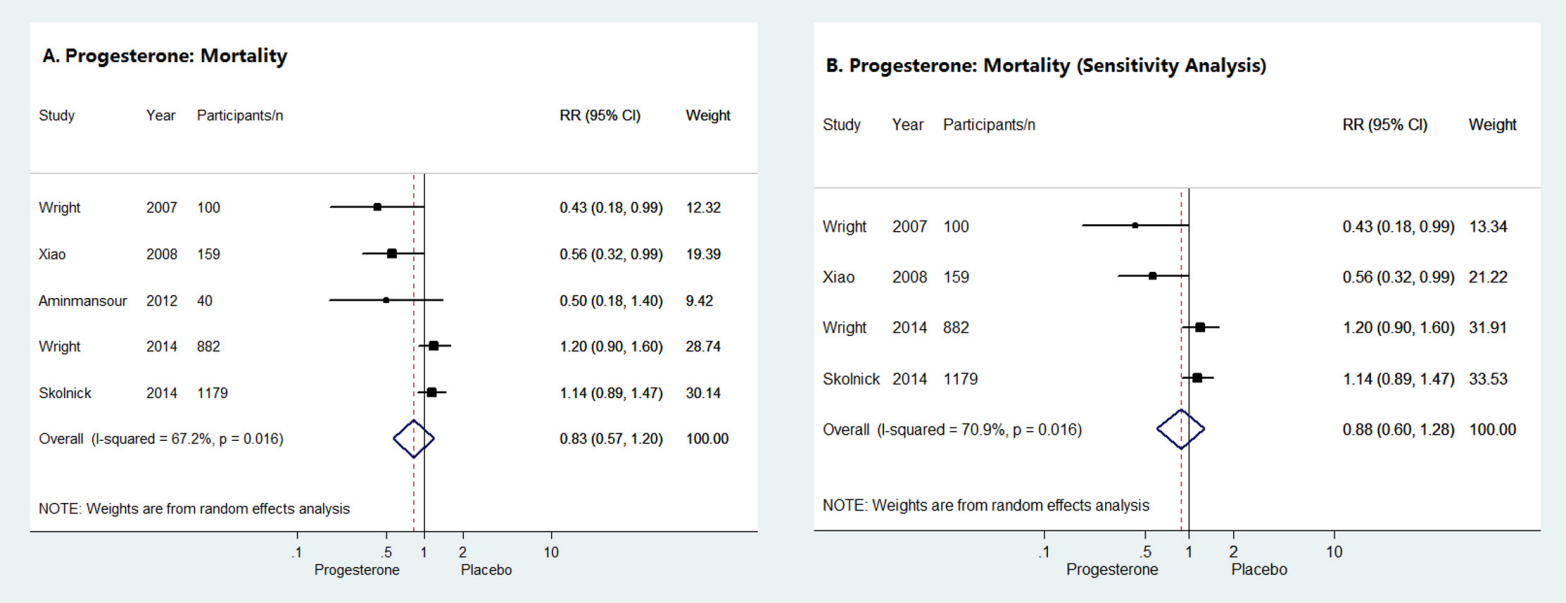
Mortality at the end of follow-up period. Forrest plots of meta-analysis of mortality for progesterone compared with placebo administrated to acute TBI patients (**A**) and sensitivity analysis of the impact of progesterone on the mortality of acute TBI patients (**B**).

Five of the six studies provided sufficient data to evaluate the impact of progesterone on the mortality of acute TBI patients at the end of follow-up period. As shown in Fig 2A, the pooled RR of the 5 RCTs suggested no difference in mortality between progesterone group and placebo group (RR = 0.83, 95% CI = 0.57–1.20), which was confirmed by the results of sensitivity analysis (RR = 0.88, 95% CI = 0.60–1.28) (Fig 2B).

### Secondary Outcome: Unfavorable Outcomes at the End of Follow-up Period

**Fig 3:**
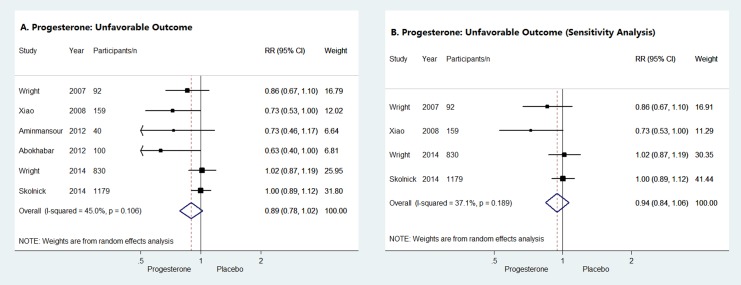
Unfavorable outcomes at the end of follow-up period. Forrest plots of meta-analysis of unfavorable outcomes for progesterone compared with placebo administrated to acute TBI patients (**A**) and sensitivity analysis of the impact of progesterone on unfavorable outcomes of acute TBI patients (**B**).

Unfavorable outcomes at the end of follow-up period were evaluated with GOS/GOS-E in all of the 6 studies of patients with acute TBI. As shown in Fig 3A, the pooled RR indicated no difference in unfavorable outcomes between progesterone group and placebo group (RR = 0.89, 95% CI = 0.78–1.02). Similarly, sensitivity analysis also suggested that progesterone did not reduce the unfavorable outcomes of acute TBI patients (RR = 0.94, 95% CI = 0.84–1.06) (Fig 3B).

### Secondary Outcome: Adverse Events for Progesterone

Four of the six studies reported the adverse events [11, 12, 18, 19]. The incidence of adverse events in progesterone group was basically equivalent to that in placebo group with the exception of phlebitis or thrombophlebitis, which was significantly more frequent in progesterone group than in placebo group in one study (17.2% vs. 5.7%, RR = 3.03, 95% CI = 1.96–4.66) [19]. The phlebitis was frequently considered as non-serious event and was self-limited. In another study, superficial phlebitis at the intravenous site was also observed in a single case which was the only adverse event attributed to progesterone and resolved spontaneously [11]. Besides, no more difference in adverse events was reported between progesterone group and placebo group. Based on the present records of adverse events, administrating progesterone to TBI patients was considered to be well tolerated and generally safe, despite of a higher risk of phlebitis.

### Evaluation of Publication Bias

**Fig 4:**
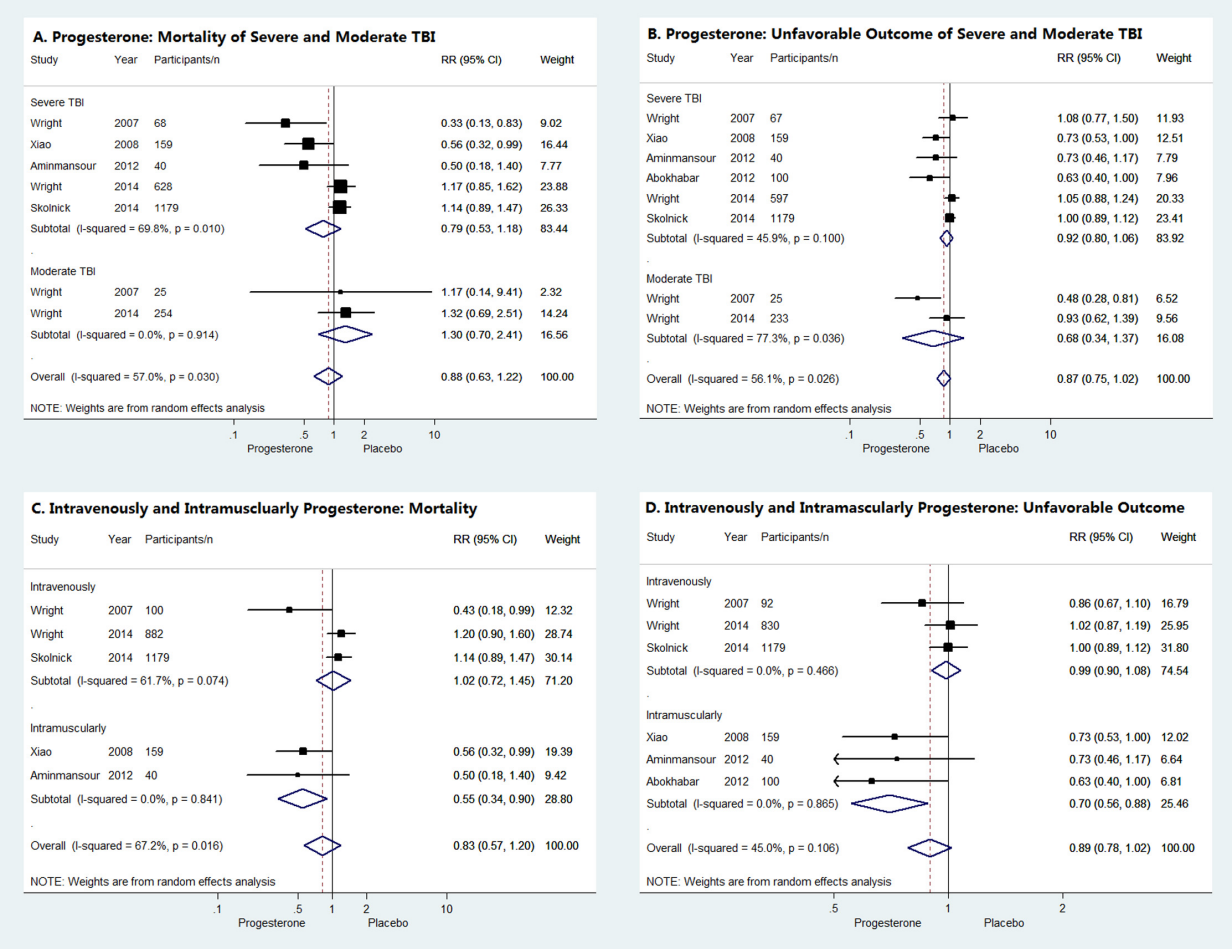
Publication bias tests. Begg’s funnel plots and Egger’s publication bias plots of meta-analysis of mortality (**A**, **B**) and unfavorable outcomes (**C**, **D**) for progesterone compared with placebo administrated in TBI patients.

Although the number of included studies was quite limited, both Begg’s test and Egger’s test were performed. No significant publication bias was detected by Begg’s test in the meta-analysis for the impact of progesterone on mortality (p = 0.806) or unfavorable outcomes (p = 0.060). However, potential bias was detected by Egger’s test (p = 0.022 and p = 0.005 for meta-analysis of mortality and unfavorable outcomes, respectively). The plots of Begg’s test and Egger’s test were provided in Fig 4.

### Subgroup Analysis

**Fig 5:**
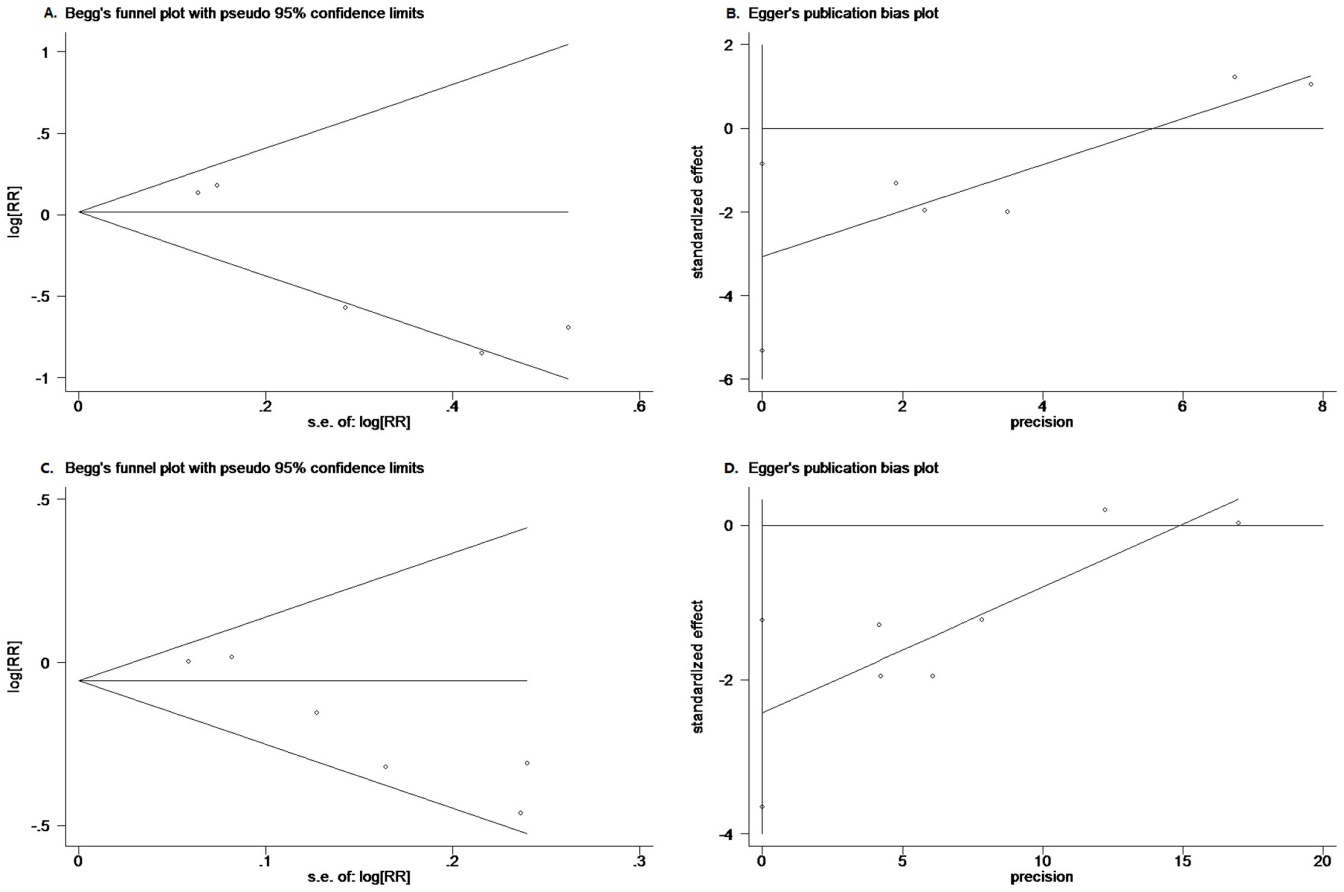
Subgroup analysis. Forrest plots of subgroup analysis according to TBI severity (**A**, **B**) and therapeutic regimens (**C**, **D**).

According to the severity of TBI, the subgroup analysis suggested neither moderate nor severe TBI patients could benefit from progesterone administration, because the mortality or unfavorable outcomes did not differ significantly between progesterone group and placebo group as shown in Fig 5A and 5B. When stratified by therapeutic regimens, intravenously administrated progesterone did not reduce the mortality or unfavorable outcomes of acute TBI patients while beneficial effect of progesterone was observed in acute TBI patients when administrated intramuscularly (RR = 0.55, 95% CI = 0.34–0.90 for mortality and RR = 0.70, 95% CI = 0.58–0.88 for unfavorable outcomes, respectively) (Fig 5C and 5D).

## Discussion

### Efficacy and Safety of Progesterone for acute TBI

Despite of the rapid development of diagnostic and treatment techniques during the past decades, TBI remains one of the leading causes of mortality and disability in both developing and developed societies. In order to find out some promising pharmacotherapy to improve the survival and recovery of TBI patients, a plenty of chemicals has been investigated with various animal models and also in some clinical trials, but no one truly effective candidate has been identified and applied to clinical practice yet [30]. The treatment of TBI remains great challenge worldwide.

More than 50 preclinical studies have been conducted to evaluate the impact of progesterone on TBI, providing considerable evidence that progesterone exerts neuroprotective function via different mechanisms [30]. Regardless of few different voices, progesterone was believed to deserve for clinical trials to assess its efficacy and safety properties in human beings. The results of early single-center studies indicated the administration of progesterone in TBI patients was both effective and well tolerated, which was really exciting [11, 12]. However, the two recently published phase III multicenter RCTs unexpectedly turned out disappointed [18, 19]. Furthermore, the results of meta-analysis and sensitivity analysis in our systematic review consistently proved that progesterone did not reduce the mortality or unfavorable outcomes of patients with acute TBI. Although the subgroup analysis of intramuscular progesterone administration seemed to get some optimistic results, it was unreliable. For one thing, only 3 small RCTs used intramuscular progesterone, which was too few to provide evidence of high quality, especially when 2 of them had high risk of bias [13, 14]. For another, the benefits of progesterone in the third RCT were quite farfetched because of the borderline RRs for both mortality and unfavorable outcomes [12].

The previous Cochrane systematic review published in 2012 only included 3 small single-center studies and its conclusions were outdated because more RCTs were carried out since then [17]. This study has updated the conclusions and confirmed the futility of progesterone in TBI patients. Mortality is the most common parameter to calculate the death of patients objectively, while GOS/GOS-E is the most common used scale to evaluate the functional recovery and handicapped degree of TBI patients. Unfavorable outcomes (GOS 1 to 3) include death which creates a sort of redundancy with mortality, but mortality and GOS/GOS-E focus differently and are equally important to estimate the conditions of TBI patients. We therefore chose these parameters, as well as adverse events, to evaluate the efficacy and safety of progesterone in the systematic review. Several indirect parameters such as blood pressure, temperature and ICP were also compared in some studies but were not included in the meta-analysis. The mean ICP of TBI patients receiving progesterone seemed to be lower than that of patients receiving placebo but the difference was not significant, which supported the results of the systematic review [11, 12, 18].

Besides the disappointing results, the failure of the two phase III trials also raised great concerns of researchers in this field and many reasons were postulated. For one thing, the treatment effect of progesterone might be overestimated in previous single-center clinical trials although the results of preclinical studies were mostly promising. As described by Ioannidis JP, many published positive research findings are probably false because of unrecognized bias and the low odds of a true relationship existing before the start of the research study [31]. Both of the two phase III trials were based on the results of preclinical studies and two phase II trials with small sample sizes [11, 12]. Nevertheless, the benefits of progesterone in the phase II trials were so modest that the statistical significance could disappear when reanalyzed by other appropriate statistical methods like Fisher’s exact test [20]. The high risk of false positive findings could result in the failure of the phase III trials. For another, although the two phase III RCTs were well designed and properly performed, some limitations still existed and could not be avoided completely, including the complexity and variability of TBI, heterogeneity of participants and insensitivity of outcome measures [18, 19]. Both trials used GCS scores to select and stratify participants, which was inadequate to characterize patients due to the complexity and variability of TBI. Meanwhile, GOS/GOS-E alone was not sensitive enough to assess the functional outcomes. Both the heterogeneity of participants and insensitivity of outcome measures could cover the true impact of progesterone on TBI. So, in addition to more rigorous statistical analysis, multidimensional approaches to characterization of TBI and reliable biomarkers predicting outcome of TBI are necessary to validate the real treatment effect and eliminate the interference of false positive findings before launching large phase III clinical trials.

Beyond the lessons learned from these RCTs, it is necessary to rethink the role of progesterone in central nervous system. Although no benefit of progesterone was proved in the treatment of patients with TBI, the potential neuroprotective properties confirmed in preclinical studies deserve more investigation. Besides adult patients with TBI, progesterone was also expected to provide benefit in pediatric TBI and other forms of brain injury such as stroke, intracerebral hemorrhage, epilepsy and other neurological diseases [32–39]. As multiple signal pathways are involved in the secondary cascades of TBI, combination of treatment targeting various mechanisms may work better than monotherapy. It is worth noting that progesterone was reported to be more effective in treatment of TBI when administrated with Vitamin D than progesterone given individually [14, 40, 41]. Progesterone in combination with other chemicals, such as progesterone with nicotinamide, magnesium sulfate and thyrotropin releasing hormone (TRH), was also investigated on animal models of TBI and better efficacy was observed [42–44]. Given the existing evidence that progesterone administration is well tolerated and generally safe, the neuroprotective potential of progesterone and progesterone combined with other chemicals can and should be explored continually in future after comprehensive preclinical studies.

### Limitations

Although systematic review is considered to provide evidence of gold standards, some limitations still exist and cannot be eliminated completely. First of all, the number of included studies in the systematic review was limited and the quality of the included studies was not as good as expected according to the quality assessment. Only 2 of the 6 RCTs evaluated the progesterone administration in patients with moderate TBI, which was insufficient to generate evidence of high quality [11, 18]. Four of the six included studies were judged to be of high quality while the risk of bias in the remaining two studies was high for several defects as described in Table 2. However, the results of sensitivity analysis were consistent with those of the overall meta-analysis, suggesting the bias in the included studies seems unlikely to make big difference in the results of this systematic review. Given that moderate and severe TBI patients were extracted from single studies, the subgroup analyses according to TBI severity were exploratory. The results were for reference only and should be explained with caution.

Secondly, in addition to statistic heterogeneity detected by χ^2^ test, the clinical heterogeneity among the 6 RCTs should also be considered, which included the difference in the severity of TBI patients, the dose, route and duration of progesterone administration and follow-up period. The heterogeneity could weaken the strength of evidence produced by meta-analysis. Besides, possible publication bias was detected by Egger’s test in the meta-analysis for impact of progesterone on mortality and unfavorable outcomes while no bias was detected by Begg’s test. This inconsistency was considered to be attributed to the limited number of included studies.

Thirdly, only published studies with available data were included in this systematic review so that some unpublished data might influence the results. Considering the fact that unpublished studies were mostly those with negative results, unpublished data might only strengthen rather than alter the negative results of this systematic review [45]. According to our retrieval results, one randomized placebo controlled trial of progesterone with or without hypothermia in patients with severe TBI was excluded for lack of detail data to perform meta-analysis, whose preliminary results revealed that progesterone group had the worst GOS outcomes while the hypothermia group had the best outcomes at 6 months after injury [16]. The preliminary results were consistent with this systematic review. The other excluded study was an ongoing RCT which was in recruiting phase in Iran with a registration date of August 21, 2014 [29]. This study was designed to evaluate the efficacy and probable mechanism of estrogen and progesterone on the complication of male patients with moderate and severe diffuse TBI. Given the target sample size of 90 patients, the influence of this single-center clinical trial on the conclusion of this review would be quite limited.

## Conclusions

In summary, despite of some modest bias, present evidence from this systematic review demonstrated that progesterone was well tolerated but did not reduce the mortality or unfavorable outcomes of adult patients with acute TBI. It is not suggested to administrate progesterone as routine treatment in patients suffering acute TBI.

## Supporting Information

S1 File PRISMA Checklist. (DOC)

S2 File Separated figures II-IV. (ZIP)
