# Supplementary figures and images for: Progesterone for Acute Traumatic Brain Injury: A Systematic Review of Randomized Controlled Trials
Source: PLoS One. 2015 Oct 16;10(10):e0140624. doi: 10.1371/journal.pone.0140624 (PMC4608716; doi:10.1371/journal.pone.0140624)

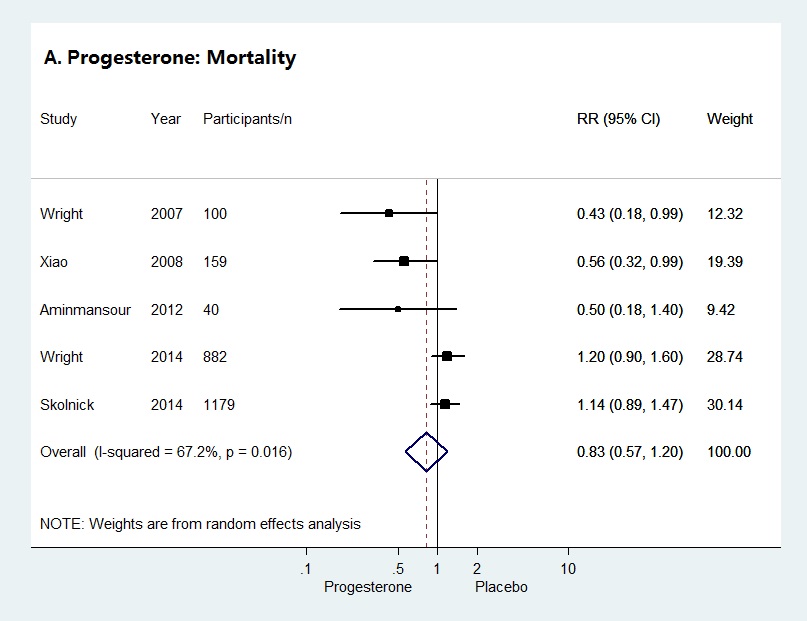

Supplement: S2 File — (ZIP) [file pone.0140624.s002.zip › Separate figures II-V/II-A.jpg]

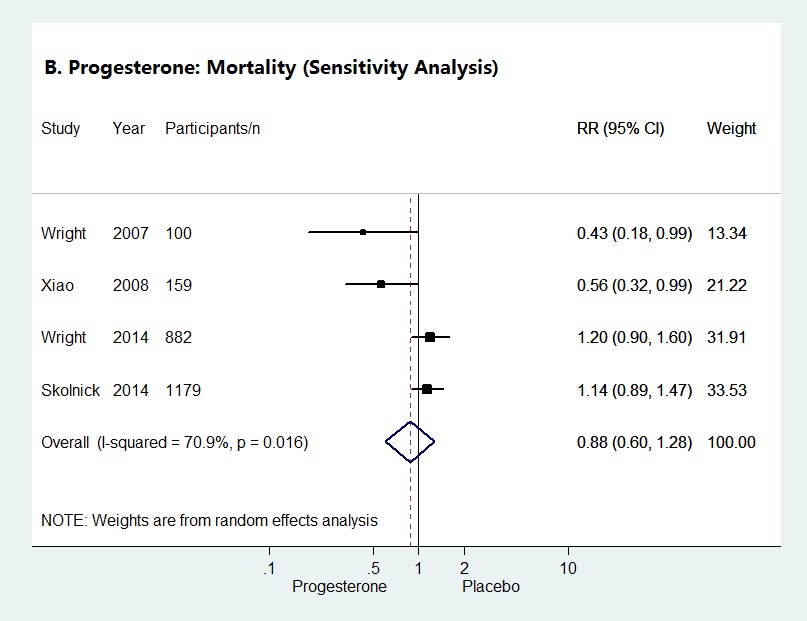

Supplement: S2 File — (ZIP) [file pone.0140624.s002.zip › Separate figures II-V/II-B.jpg]

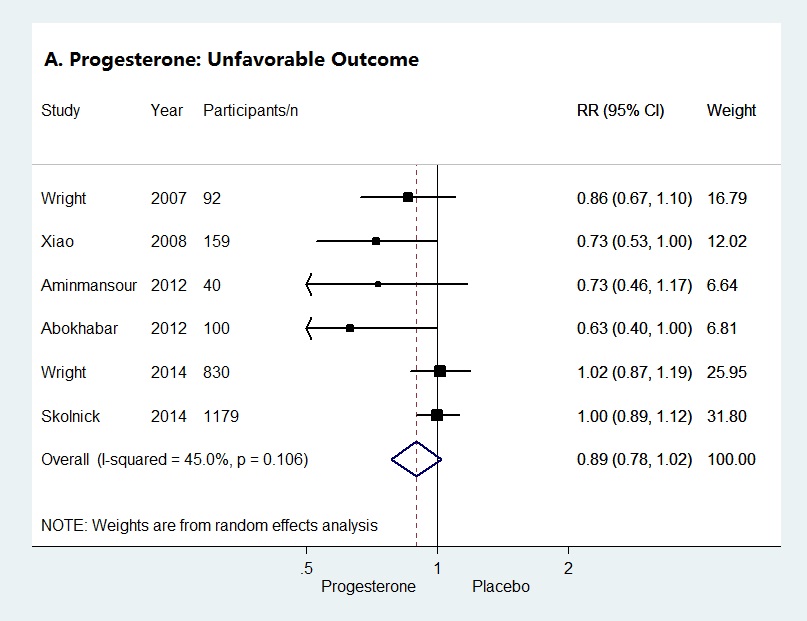

Supplement: S2 File — (ZIP) [file pone.0140624.s002.zip › Separate figures II-V/III-A.jpg]

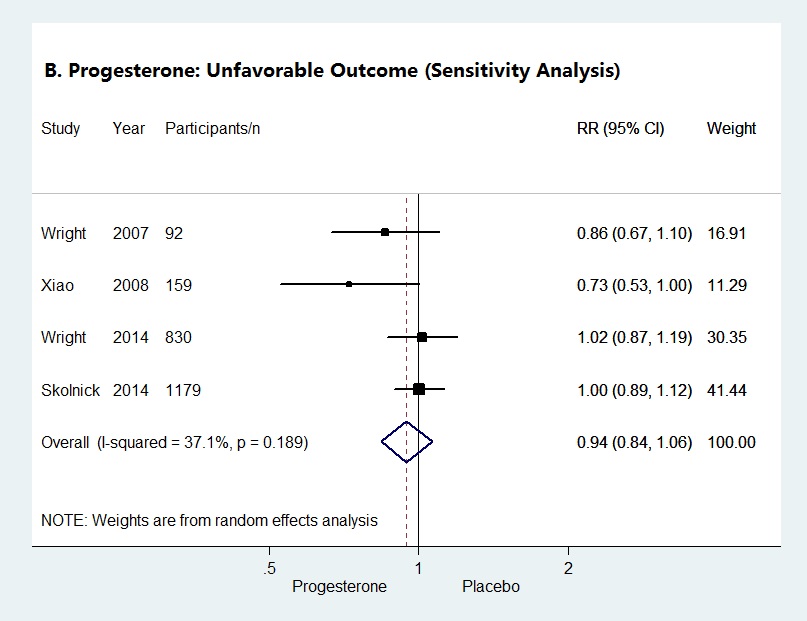

Supplement: S2 File — (ZIP) [file pone.0140624.s002.zip › Separate figures II-V/III-B.jpg]

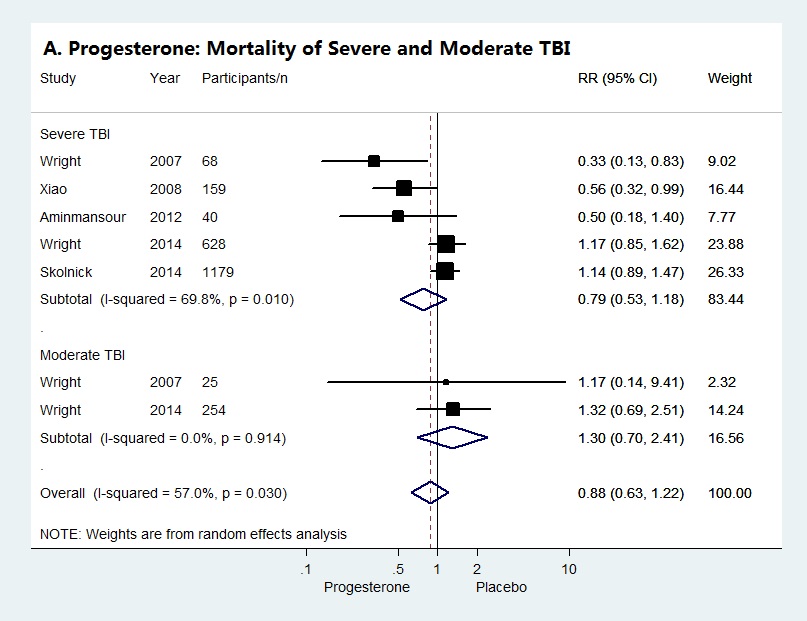

Supplement: S2 File — (ZIP) [file pone.0140624.s002.zip › Separate figures II-V/IV-A.jpg]

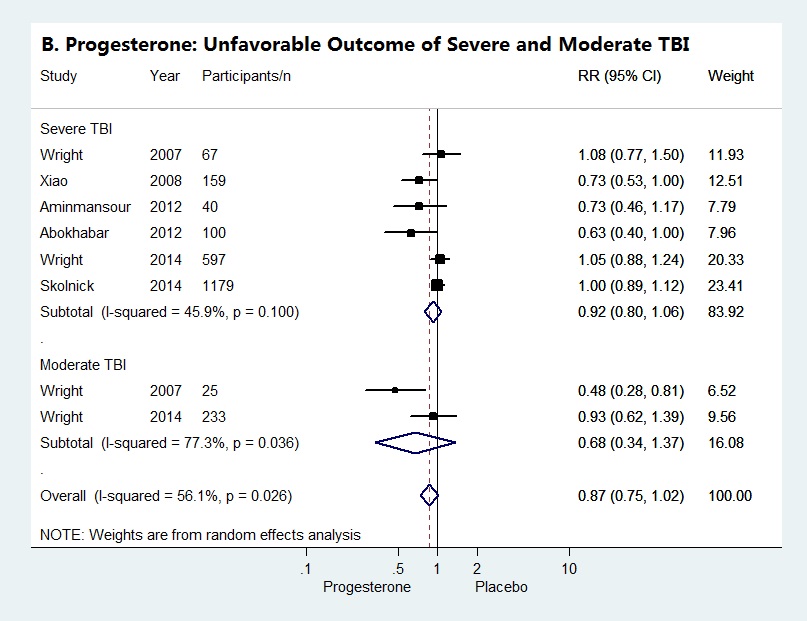

Supplement: S2 File — (ZIP) [file pone.0140624.s002.zip › Separate figures II-V/IV-B.jpg]

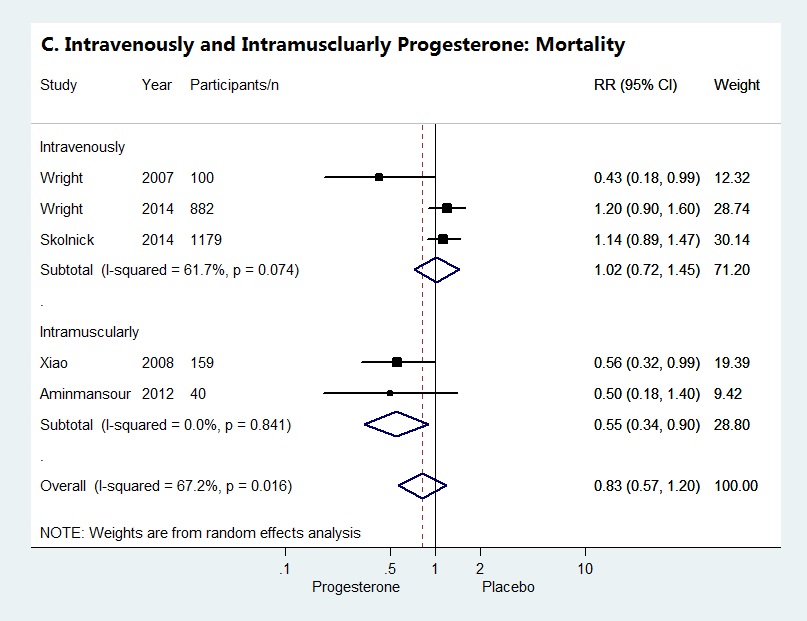

Supplement: S2 File — (ZIP) [file pone.0140624.s002.zip › Separate figures II-V/IV-C.jpg]

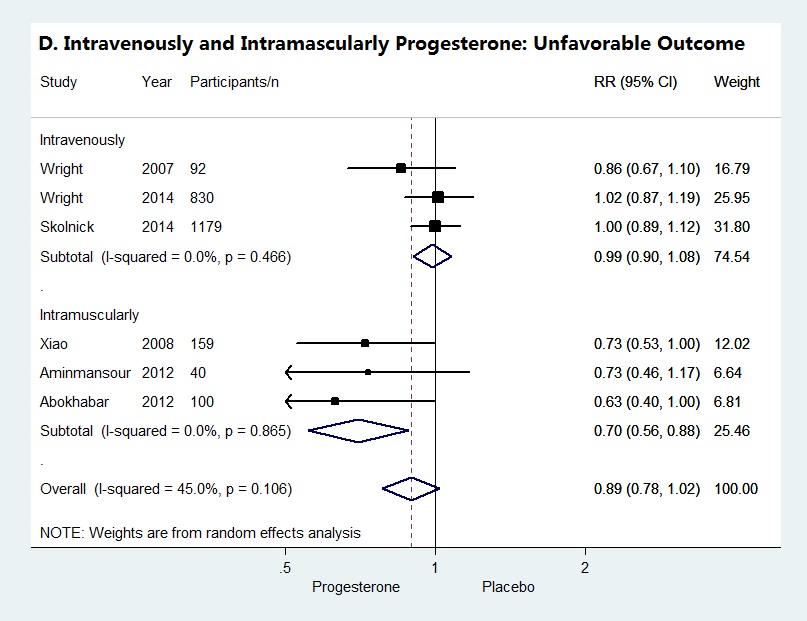

Supplement: S2 File — (ZIP) [file pone.0140624.s002.zip › Separate figures II-V/IV-D.jpg]

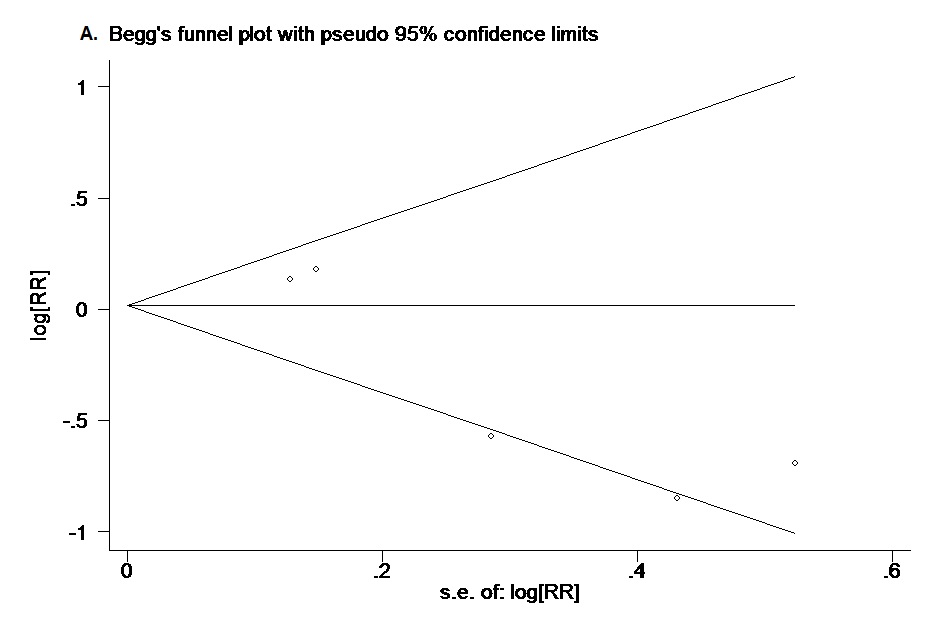

Supplement: S2 File — (ZIP) [file pone.0140624.s002.zip › Separate figures II-V/V-A.jpg]

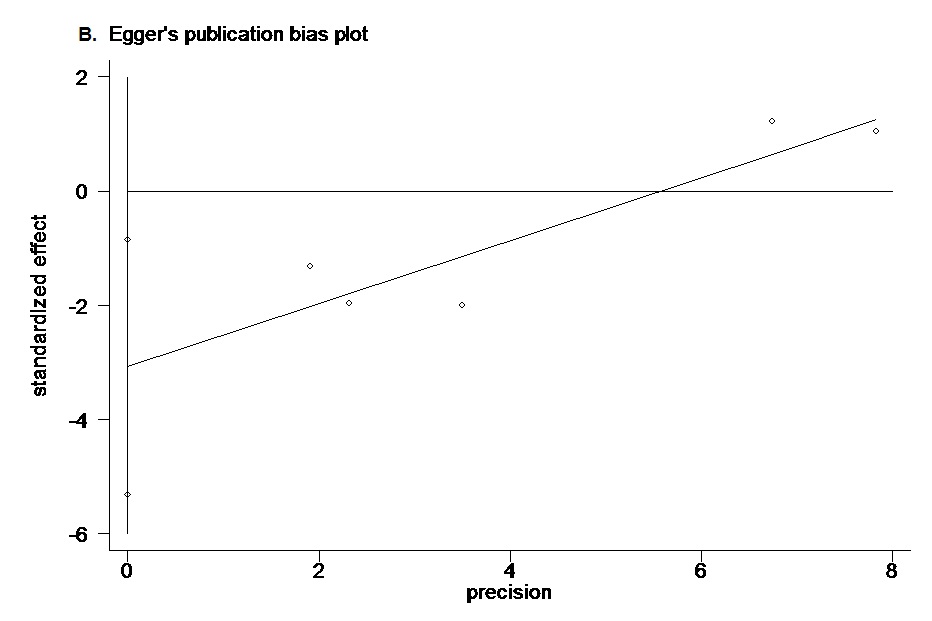

Supplement: S2 File — (ZIP) [file pone.0140624.s002.zip › Separate figures II-V/V-B.jpg]

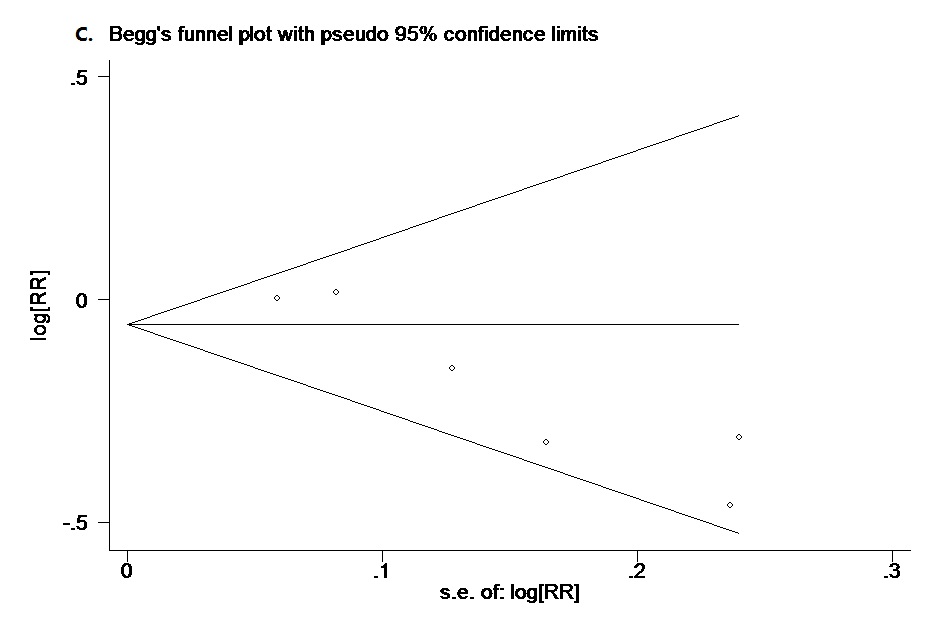

Supplement: S2 File — (ZIP) [file pone.0140624.s002.zip › Separate figures II-V/V-C.jpg]

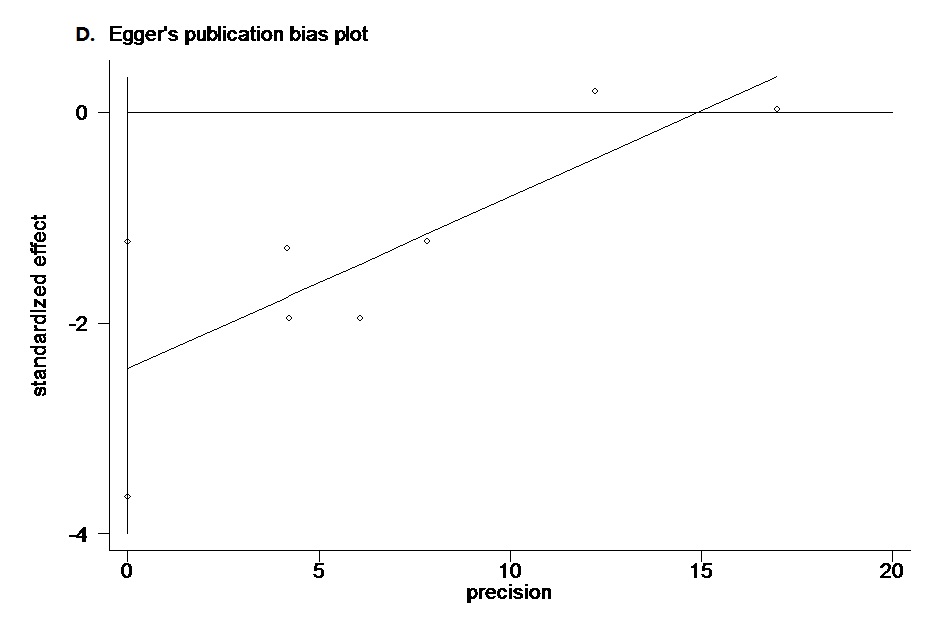

Supplement: S2 File — (ZIP) [file pone.0140624.s002.zip › Separate figures II-V/V-D.jpg]
